# An Updated Overview of Silica Aerogel-Based Nanomaterials

**DOI:** 10.3390/nano14050469

**Published:** 2024-03-04

**Authors:** Adelina-Gabriela Niculescu, Dana-Ionela Tudorache, Maria Bocioagă, Dan Eduard Mihaiescu, Tony Hadibarata, Alexandru Mihai Grumezescu

**Affiliations:** 1 Research Institute of the University of Bucharest—ICUB, University of Bucharest, 050657 Bucharest, Romania; adelina.niculescu@upb.ro; 2 Department of Science and Engineering of Oxide Materials and Nanomaterials, University Politehnica of Bucharest, Gh. Polizu St. 1-7, 060042 Bucharest, Romania; dana.tudorache@stud.fim.upb.ro (D.-I.T.); maria.bocioaga21@gmail.com (M.B.); hadibarata@curtin.edu.my (T.H.); 3 Department of Organic Chemistry, Politehnica University of Bucharest, 011061 Bucharest, Romania; danedmih@gmail.com; 4 Environmental Engineering Program, Faculty of Engineering and Science, Curtin University, Miri 98000, Malaysia

**Keywords:** silica aerogel, aerogel preparation, aerogel properties, aerogel-based composites, applications

## Abstract

Silica aerogels have gained much interest due to their unique properties, such as being the lightest solid material, having small pore sizes, high porosity, and ultralow thermal conductivity. Also, the advancements in synthesis methods have enabled the creation of silica aerogel-based composites in combination with different materials, for example, polymers, metals, and carbon-based structures. These new silica-based materials combine the properties of silica with the other materials to create a new and reinforced architecture with significantly valuable uses in different fields. Therefore, the importance of silica aerogels has been emphasized by presenting their properties, synthesis process, composites, and numerous applications, offering an updated background for further research in this interdisciplinary domain.

## 1. Introduction

Among all solid materials, aerogels are considered the “miracle material for the 21st century” due to a pearl necklace-like network of combined particles resembling a large porous structure, with a volume of air of 80–99.8% [1,2]. This unique aspect gives aerogels the status of the lightest solid materials with the best insulation properties [2]. The term “aerogel” derives from the fact that they are produced by replacing the liquid constituent of a gel with a gas without disrupting the network structure [3,4]. Contrary to the implications of the name, aerogels are stiff, dry materials and do not physically resemble a gel [5]. They also exhibit a rather low apparent density, a translucent structure, a low refractive index, and an exceptionally high specific surface area [1,2,4,5,6].

Samuel Kistler made the first aerogel in the 1930s, a material that was later translated to the industry for thermal insulators and viscosity regulators [7]. In recent years, aerogel has seen significant advances and changes as a thermal superinsulation material. Its diverse appeal has led to various applications, including buildings, cars, electronics, clothing, and more. Aerogel has attracted much attention due to its exceptional chemical and physical properties, especially for energy-efficient retrofit possibilities in residential structures [5].

**Figure 1:**
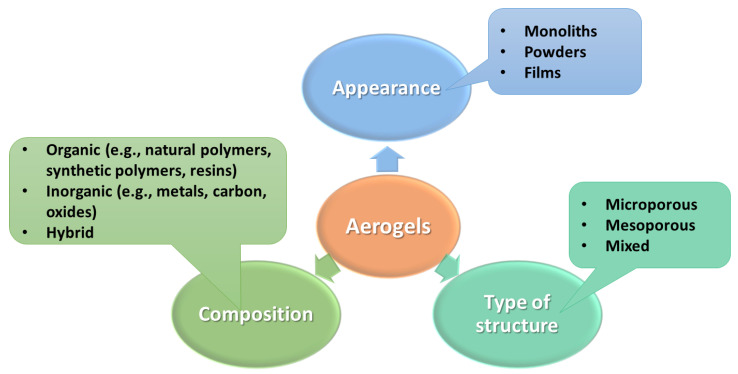
Classification of aerogels established on precursor composition. Created based on information from [9,11,12,13,14].

Aerogels can be classified in various ways, including by the preparation technique (aerogel, xerogel, cryogel, and alternative aerogel-similar materials) or by their appearance (monoliths, powder, films). Also, regarding the pore size and the pore distribution, the aerogels can have different microstructure characteristics: microporous (<2 nm), mesoporous (2~50 nm), and mixed-porous aerogels [8,9,10]. Aerogels can also be hybrid, inorganic, or organic, as represented in Figure 1 below [8]. However, identifying aerogels based on their composition is the most appropriate classification. According to this perspective, there are two main categories of aerogels: aerogel composites and single-component aerogels. The single-component class includes oxide aerogels (non-silica or silica), organic aerogels (cellulose-based and resin-based), carbon aerogels (carbon nanotube, graphene, and carbonized plastic), chalcogenide aerogels, and additional kinds of aerogels (carbide, single element, etc.). Aerogel composites involve gradient aerogels, micro- or nano-aerogel composites, or multi-composition aerogels [9,10].

Aerogels can be created from a wide range of materials, such as metal oxides, polymers, and biopolymers, although silica aerogels are by far the most popular [4,15]. Compared to another highly investigated class of aerogels (i.e., graphene aerogels), silica-based materials can be obtained through less expensive and more environmentally friendly drying processes [16]. In contrast to biopolymeric (e.g., cellulose) aerogels, silica-based aerogel structures present better water and fire suppression properties [17].

End uses of silica aerogels that have been reported include adsorbents of harmful compounds [18,19], sensors [20], conductive and dielectric materials, gas filters and storage materials of gases [21], Cherenkov detectors [22], kinetic energy absorbers [23], catalysts and catalyst support [21,24], extraction agents [25,26], and protective clothing [27]. However, there is no doubt that the most important uses of silica aerogels are in thermal and acoustic insulation [28,29], with numerous in-depth reports in this field [30,31,32,33].

Although nanoscale porosity provides unique features, silica aerogels are not widely used in everyday applications, mainly due to their intrinsic fragility, which makes handling and processing difficult. Expanding the application of such promising materials will require overcoming other intrinsic disadvantages such as dust release, hydrophilicity, volumetric shrinkage, long processing time, and, last but not least, the prohibitive cost of silica aerogels, which are 8–20 times more expensive than polyurethane foam [2,31,34,35,36].

In this context, this paper presents silica-based aerogels in a comprehensive manner. Specifically, the preparation methods, properties, composites based on silica aerogels, and the applications of these versatile materials are described to create an updated framework for future research in the field. By describing the state-of-the-art silica-based aerogels and underlining the remaining challenges, this review aims to serve as an inception point for future studies, encouraging technological advancements through an interdisciplinary perspective.

## 2. Preparation Methods

Aerogel synthesis begins with the hydrolytic formation of primary nanoparticles or building blocks, which are then condensation-formed into a network structure. Then, the liquid trapped within the pores of the system is eliminated without collapsing the network structure. During the synthesis process, the conflicting hydrolysis and condensation processes occur. Furthermore, drying and network development are the two most important processes during the synthesis of aerogels [37]. Table 1 presents different techniques for aerogel manufacture, highlighting their advantages and disadvantages.

**Figure 2:**
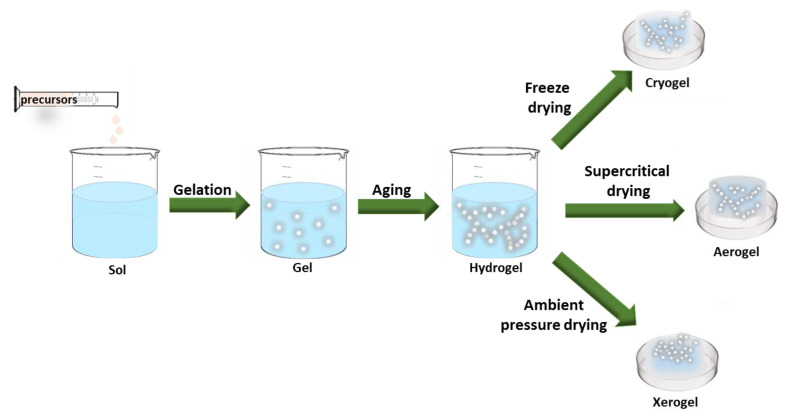
The stages of gel formation. Created based on information from [56,57,58].

Due to their significant properties, in recent years, silica aerogels, an ultralight inorganic, have gained great attention for their use in different technological applications [42,52]. The synthesis of silica aerogels can be divided into three general steps: (1) gel preparation by sol-gel processes in which nanoscale sol particles are spontaneously formed in the precursor solution or catalyzed by catalysts through hydrolysis and condensation reactions [6,9,53], (2) aging the gel in its mother solution to prevent gel shrinkage during drying, and (3) drying the gel under special conditions to prevent the destruction of the gel structure. Control of the microstructure during preparation is crucial, as all three phases can potentially affect the aerogel’s microstructure, properties, and uses [5,9,54,55]. Figure 2 shows the schematical illustration of the three steps mentioned: preparation, aging, and drying techniques.

### 2.1. Silica Aerogel Synthesis

#### 2.1.1. Gel Preparation

Sol-gel preparation is a process in which the colloidal particles are dispersed together to form a tridimensional network that expands through the solution volume [37]. Aerogels fundamentally represent the solid framework of a gel that was set apart from its liquid form. In the case of silica aerogels, nanoparticles are directly grown in the liquid [54]. For the sol-gel process, the precursor represents the first material for the start, and it should be capable of disintegration in the reaction medium; at the same time, it is necessary to be reactive enough to take part in the form of the gel process. Precursors that are soluble in appropriate solvents can be salts, amines, acylates, oxides, complexes, hydroxides, and alkoxides [59]. Silicon alkoxides are the most widely used precursors for sol-gel silica chemistry due to their versatility and almost easy chemistry [2]. Some examples of the most used silica precursors are tetramethoxysilane (TMOS) with the chemical formula Si(OCH_3_)_4_, tetraethoxysilane (TEOS), Si(OC_2_H_5_)_4_, or polyethoxydisiloxane (PEDS-Px), SiO_n_(OC_2_H_5_)_4−2n_, n < 2 [54].

The selection of the precursor is an important step because it helps control the final properties of the material, leading to products with custom-made chemical and physical features. In general, silica aerogels can be divided into three classes acquired from pure silica, organically modified silicas (ORMOSILs), and organic–inorganic composite aerogels [13].

The component from which the aerogel is hydrolyzed and synthesized is a catalyst, which can be acid, base, or a two-step [5,54,57]. During the gel preparation phase, the solid nanoparticles spread into it and adhere together to build a medium of particles covering the liquid. The condensation and the acid hydrolysis in silica sols result in frail branched or linear chains and microporous structures, and the gelation times are long in most cases. In base catalysis (i.e., NH_4_OH-based mostly), the uniform particles are effortlessly formed and tend to create large pores with a broader distribution [5,54]. In essence, in the sol-gel method, the first step is the preparation of a precursor of the colloidal matrix in a sol(liquid). Next is the gel manufacture, a porous solid stage where the porosity can be tailored by balancing the condensation and hydrolysis rate by settling different parameters, such as pH and the chemical composition of the specific precursors and temperature [2,57].

#### 2.1.2. Aging

Even after the gelation point has been attained, the sol-gel chemical reaction still exists because the liquid within the pores has small particles that may condense or roving monomers that can attach to the network. The term “aging” represents the crosslinking continuous process and coarsening as a result of additional condensation [2]. These two reactions—hydrolysis and condensation—may persist, and they need enough time for the silica network to become stiff and strong by controlling the concentration, water content of the enclosing solution, and pH [5,54,55].

During the aging process, two dissimilar mechanisms might have an impact on the properties and the structure of the gel: neck growth from the chemical reaction of reprecipitation of silica dispersed from the surface of the particle onto necks among particles (either polymeric bridge or siloxane link) and production of broad particles by precipitation and dissolution of the little particles [5,42,53,54]. The aging is controlled by diffusion: the material transport is unchanged by convection or associating due to the silica network, which is solid, even though the gel thickness influences diffusion itself. As a consequence, the necessary time for each processing step grows as the gel thickness increases, restricting the aerogels’ practical production [54].

All the remaining water within the pores, after the aging, must be taken off before the next step, the drying process. All the remaining water can be easily removed by washing the gel with heptanes or ethanol. If the water is still in the gel, by supercritical drying, the aerogel will be very dense and opaque [5,54].

#### 2.1.3. Drying

The last step of the sol-gel method is represented by the drying process. This method is based on the elimination of the solvent from the matrix framework without causing related capillary forces and a two-phase system that generates partial or total loss of the nanostructure of the gel. If the removal of the liquid phase was executed in a non-destructive way, it would yield a porous solid with an unaffected shape and volume as the initial gel [2,60]. Drying, as with the composition of the gel, prescribes pore dimension and textural properties, making it a critical step in the manufacturing aerogel process. Therefore, it is necessary to choose a method that has a big relevance. There are various drying techniques with pros and cons shown in Table 2, such as supercritical drying, ambient pressure drying, freeze-drying, microwave-drying, and vacuum drying. Still, the most used of them are ambient pressure and supercritical drying [2,5,54,61].

##### Supercritical Drying (SCD)

Supercritical drying was first invented in the 1930s when the first aerogels were manufactured, and since then, it continued to be the most well-known drying method to obtain a lower-density aerogel. In supercritical drying, the drying of gels happens, as the name implies, at a critical point to clear up the capillary forces since the interface of the liquid gas is suppressed [2,5,48]. Even though this process is one of the most used techniques, it has two main disadvantages, such as the huge complexity of the machinery required to function at high pressure and the considerable cost [2,18,35].

**Figure 3:**
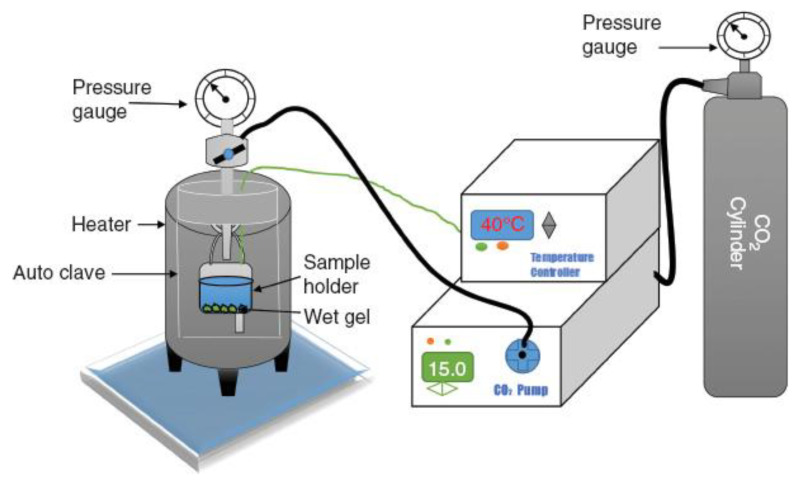
Schematic illustration of supercritical drying device. Reprinted from an open-access source [72].

Supercritical drying involves the positioning of a solvent-rich gel in a closed container and exposed to a gas, such as carbon dioxide (CO_2_) or methane, with the purpose that the temperature and pressure in the reactor are assisted above critical points of the solvent embedded in the gel pores [44,61]. As the pressure and temperature in the autoclave grow over a critical point, the surface tension is eliminated because the liquid becomes a supercritical fluid where every molecule is capable of moving voluntarily [5]. Figure 3 shows the schematic illustration of supercritical drying equipment.

The supercritical drying technique can be divided into two different methods: low temperature (LTSCD) and high temperature (HTSCD) [53,54]. In LTSCD, organic solvents from the gel, like methanol, acetone, and ethanol, can be substituted by soluble CO_2_, which will transform into supercritical carbon dioxide (Sc-CO_2_). This transformation can happen exclusively at a critical temperature close to the room’s temperature and finally attends to the creation of gel [61]. In HTSCD, the substitution of hydrogel with an organic solvent, such as the ones enumerated previously, is necessary, and after that, it is inserted in a container for pressurization and heating. The solvent discharges out from the gel when the supercritical state of the solvent is reached. Even though this method is found to be the most convenient technique to minimize the contraction of the gel, it is not recommended to obtain aerogels for use in the construction domain [5,54,61].

##### Ambient Pressure Drying (APD)

Compared to the HTSCD and LTSCD drying methods, which are expensive, ambient pressure drying is commercially attractive for silica aerogel production due to the lower cost [5,54]. APD is a favorable drying process used in industrial risings. This method acts to inactivate the pore’s surface from the wet-gel, meaning there is no switch to forming new chemical bonds during wet-gel drying [61].

Generally, ambient drying unfolds in two steps. The first step is silylation, where all the Si-OH groups are silylated, and the water adsorption is prevented, producing a hydrophobic aerogel. This is enforced by substituting the current solvent with a water-free one and a silylating agent (e.g., TMCS, HMDZ, HMDS), which ensures the substitute of H from Si-OH groups by an alkyl, CH_3,_ for example. The ambient pressure evaporation represents the second step and can be further separated into three stages. After a heating period, the first drying stage occurs, where the volume deficit of the gel and liquid that was evaporated are balanced. Then, the liquid flows to the external surface straight from the partially empty pores because of the capillary forces during the first falling rate period. The second falling rate stage is when the liquid slowly withdrawals from the drying gel via the transport of diffusive vapor to the exterior [53,54].

##### Freeze-Drying

Freeze-drying, also called lyophilization, is another option for drying aerogels. This method is environmentally friendly, simple, and economical, and it can obtain aerogel with reduced shrinkage and high porosity [2,5,61]. In this procedure, the liquid inside the pores is frozen and sublimed in a vacuum under low pressure [5,61].

The lengthy aging period to stabilize the gel network is one of the problems associated with this method. One with a lower expansion coefficient and a higher sublimation pressure shall also be substituted for the solvent [5]. This method is used in the dehydration of hydrogels because the solidification of water is more easily available than that for conventional solvents [2].

Three steps are part of the lyophilization cycle: first, the temperature of the solvent inside the sublimation pores is lowered below the triple point; second stage, the system is evacuated until vacuum; and the final step is controlled under isobaric conditions [2].

##### Microwave-Drying

One of the efficient ways to prepare 3D interconnected mesoporous and microporous structures is to use the microwave-drying strategy because it saves time. This method produces aerogels that are almost identical to the resulting aerogel by means of freeze-drying, but the formed macropores are very small [61].

The principle of microwave-drying is based on fast fine-drying in an oven and avoiding high temperatures. By utilizing the alternative electromagnetic field, an aspect of the microwave, the liquid molecules begin to spin and align their electric dipoles. This resulted in a growth in the global molecule vibration, promoting the molecules’ own heating. Compared with evaporative drying in the oven, microwave drying has some advantages, such as the heat being internally created within the material, which facilitates the reduction in thermal gradients and cracking that may occur during the drying process; the time and energy consumption for this process are reduced [70,73].

##### Vacuum Drying

In the course of developing aerogels for many years, microwave and vacuum drying methods were used to dry the wet gel to obtain an aerogel with a high specific surface area and a large porous structure [61].

In contrast to alternative drying techniques, vacuum drying offers enhanced convenience for surface modification, reducing the necessary drying time [74]. Due to the use of a shorter amount of heat, compared with traditional supercritical drying, this technique has been designed for energy savings and time-saving without requiring specialized equipment for high pressure, surface modification, or challenging solvent exchange. Under reduced pressure, vacuum drying techniques have been applied. Microporous aerogels have been prepared with a high surface area and a narrow-distributed size of less than 6Å pore dried by vacuum [61,75].

### 2.2. Modification/Functionalization Methods

Materials made from wet gels often have final hydrolyzed groups (M-OH) covering both their interior and exterior surfaces. The hydroxyl groups from the surface can be modified with alternative functional groups using precursors with hydrolyzable molecules containing the appropriate functionality. Through this technique, organic or inorganic moieties can be integrated in a controlled manner, including other nanoelements such as nanotubes, nanofibers, particles, etc. [76].

The fragile skeleton might be one of the disadvantages of aerogel. Even if aerogels are adequately robust to be managed, they remain considerably fragile materials, and for some useful applications, their mechanical strength is not suitable. Therefore, various techniques must be employed to reinforce the mechanical properties of aerogels [10].

The skeleton of aerogel can be strengthened with a polymeric system of a compound of an inorganic network. However, the novel approach that allows for a noticeable improvement in the polymer nanocomposite’s thermal and mechanical properties is using aerogel as a filler in the polymeric system. To create ultralight materials with exciting properties and many potential applications, like energy storage devices and featherweight nanocomposites, a straightforward strategy is to obtain polymer-aerogel nanocomposites [10].

However, chemical crosslinking with polymers or reactive molecules is necessary to improve the mechanical properties. Polymers-reinforced SiO_2_ can be classified into two categories: reinforced with polymer macromolecules and, the second one, with a polymer monomer. The polymer monomer method can be split into three other methods: copolymer precursor modification, solution impregnation modification, and vapor deposition modification. The polymer macromolecule method is based on the addition of polymer macromolecules in gel without the implication of a polymerization reaction. After the sol is manufactured, polymer macromolecules are directed and added to make the aerogel, followed by aging and drying. The inorganic and organic phase interface is chained by powerless interaction forces, namely van der Waals, electrostatic gravitational, hydrogen bonds, or covalent bonds that are strong. The increased mechanical qualities of silica aerogel result from the superior mechanical properties exhibited by long carbon chains in polymers [77].

The modification with copolymer precursor is based on using a polymer and silicon source as a precursor to making, through chemical reaction, silica sol, and gel. Following the reaction of sol-gel, the functional group facilitates polymerization by means of polymer chains connecting particles via weak silanol bonds. The utilization of this approach guarantees a uniform distribution of the polymer all through the aerogel skeleton [77,78].

Contrary to the method of copolymer precursor, the modification with solution impregnation supposed the impregnating silica gel or sol in the polymer solution to generate a polymerization reaction. Some examples of polymers are styrene, epoxy resin, isocyanate, methyl methacrylate, etc., which can be utilized as a solution for impregnation modification. For the polymer solution to come into touch with the pores of the wet gel, it should be submerged in the gel. Capillary pressure is generated due to surface tension; therefore, the solution becomes immersed within the wet gel, and a chemical band is stamped between silica and polymer, which advances the mechanical and additional properties [77].

Chemical vapor precipitation (CVD) is a method used to transfer a solid material, silica, for example, into a substrate through a surface reaction or a gas phase. Hence, the SiO_2_ aerogel can likewise be enhanced effectively via vapor deposition. CVD is also capable of imparting unique properties to aerogel. Solid deposits are formed when chemical reactions occur at the interface of the gaseous or vapor polymer in the reactor and the gas-phase or gas–solid medium. These reactions are predominantly fueled by various energy sources, including light radiation, heating, and plasma [77,79].

The incorporation of inorganic substances remains a viable method for enhancing the mechanical characteristics of silica aerogel. With a high degree of stability, inorganic materials are resistant to heat, acid, and alkali. Inorganic reinforcement provided aerogel composites with superior thermal stability, chemical resistance, and possibly catalytic activity. Preparation methods for inorganic reinforced aerogels include sol-gel, impregnation, and CVD [77].

## 3. Silica Aerogel Properties

### 3.1. Pore Structure

Aerogels have a unique combination of small pore size and high porosity. An important aspect of the pore network from aerogel is interconnectivity and the “open” nature. However, in a structure with an open pore, the liquids can run out with limited restriction, from pore to pore, and finally travel over the entire material [53,80].

**Figure 4:**
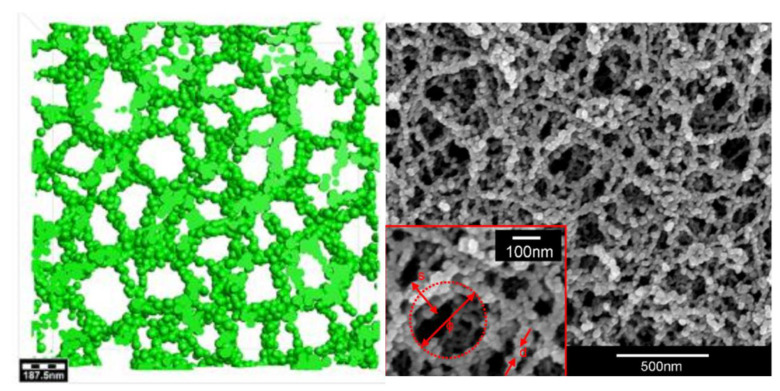
Simulated pore structure of silica aerogel (**left**) and SEM images of silica aerogel (**right**). Adapted from an open-access source [81].

Of all three aerogel types, carbon, silica, and alumina, silica aerogel is the remarkably used and investigated type due to its remarkable solid properties. Silica aerogels are made up of several air-filled pores and a crosslinked internal framework of SiO_2_ chains (Figure 4). These pores are pretty small; pure aerogels have a pore diameter between 10 and 100 nm, and silica aerogels generally can have a pore size between 5 nm and 70 nm, depending on the fabrication method and purity [5,54].

### 3.2. Density

Due to their high porosity, aerogels are known today as the lightest solid material. Two specific terms are used to characterize silica aerogels: bulk and skeletal density. The bulk density is known as the mass-to-volume ratio of the aerogel. These particles’ skeleton densities are expected to be highly similar to the bulk density. The value of bulk density ranges from 3 to 300 kg/m^3^, and the skeleton density is approximately 2200 kg/m^3^, compared with air density of approximately 1.2 kg/m^3^ [5,53,54,55,80].

Also, silica aerogels show a high compression strength of up to 3 Bar but a low tensile strength, which makes them a fragile material. Due to the surface tension inside the pores, the aerogel structure could be demolished if it is not well hydrophobized. In this situation, aerogel is frequently employed in conjunction with a vacuum, which further lowers the thermal conductivity while the envelope stops water inclusion [54].

### 3.3. Thermal Conductivity

The most studied property of silica aerogels is thermal conductivity. Due to nanometer pore size and porosity, this material is used as an isolating material because of its ultralow thermal conductivity, which is lower than still air [53,80,82]. This lower value, 0.02 W/mK in the ambient pressure and 0.01 W/mK when evacuated, results from a low gaseous conductivity, a small solid skeleton conductivity, and an inferior radiative infrared transmission [53,54,80]. The structure of silica aerogel can be defined as a mesoporous structure with a significant volume fraction of mesopores, and a randomly organized low-volume fraction is responsible for the low thermal conductivity value [83].

The thermal conductivity (*λ*_*tot*_) (W/(m K)) property is represented by the solid skeleton conductivity, gaseous conductivity (*λ*_*g*_), and radiative infrared transmission (*T*_IR_). However, it may be a challenge to calculate the general thermal conductivity by summing all components due to the close coupling of modes; for instance, a modification in the solid skeleton conductivity will also lead to an alteration in the infrared absorbance [54].

### 3.4. Hydrophobicity

Contingent upon the condition throughout synthesis, silica aerogels can be hydrophobic or hydrophilic. The primary source of hydrophilicity is the Si-OH silanol polar groups that exist in the structure of aerogel because they can advance water adsorption. Generally, hydrophobic aerogels are synthesized by alkyl orthosilicates’ unmodified hydrolysis process and condensation and dried by high-temperature supercritical drying, and hydrophilic aerogels are dried by CO_2_. The many surface groups that are created throughout the SCD process are the cause of this discrepancy [53,80,84].

There are two distinct routes to grow hydrophobicity. The hydrophobic character can be increased during the sol-gel process by adding a silylating agent, and a second approach is to modify the surface of the aerogel after drying by reaction with the methanol in the gaseous phase [53].

### 3.5. Optical Properties

The phrase “silica aerogels are transparent” is the best description of silica aerogels’ optical properties, and it may be obvious because they are manufactured from the same element as glass [80]. Because these are transparent, and for a porous material is an uncommon property, the reason for this is the aerogel microstructure, which has a small scale correlated with the light wavelength [53,84].

By heating the aerogels, due to the combustion of organic components and desorption of water, their transparency can improve. Also, the parameters of the sol-gel technique and the silation agent type greatly influence the optical properties [53].

### 3.6. Mechanical Properties

For theoretical research, a clear interest is in understanding the mechanical properties of aerogels because, in connection with the structure, they can be studied experimentally over the entire range of porosity [85]. Since aerogels synthesized under neutral or mildly acidic circumstances can be twice as stiff as aerogels generated under basic conditions, the mechanical properties of native silica aerogels and the manufacturing process are rather significant. Appropriate aging and heat treatments can also increase aerogel strength. However, despite optimized synthesis conditions, silica aerogels remain fragile due to their high porosity and ionic–covalent bonding [2].

The mechanical properties, especially yield stress and elastic modulus, provide access to designing and developing aerogels with attractive stiffness and strength [81]. The mechanical properties, especially yield stress and elastic modulus, provide access to designing and developing aerogels with attractive stiffness and strength. Also, the mechanical properties depend directly on their density, with three areas of expertise in fact of their uniaxial compression response. Silica aerogels, at low densities, are non-brittle, deform plastically, and are very compressible. They can withstand relative strain levels far beyond 50% in a controlled laboratory environment without brittle rupture. However, the deformation is almost permanent, and the materials fail to regain their initial volume once the load is removed. They become brittle at intermediate densities, and with increasing density, the deformation becomes progressively elastic and can get back most of the tension upon decompression. Silica aerogels with a high density are very brittle and certainly not as compressible as intermediate and low-density aerogels [86].

## 4. Composite Materials Containing Silica Aerogel

Even for some applications, the properties such as fragile and low-pressure sensitivity represent disadvantages. Recent evolutions in synthesis have enabled the creation of silica composite aerogels to improve properties [40]. Another important development is represented by the utilization of noble metals, like silver and gold nanoparticles, incorporated in silica aerogels via gamma-ray irradiation and ambient pressure radiolysis for application in catalytic [12].

The combination of the biodegradability of organic components and the large surface area of inorganic constituents endows novel materials with interesting properties. Hybrid aerogels, a material entrenched in both inorganic and organic components, advanced to excellent and some novel physiochemical properties [87]. Classification of hybrid aerogels into Class I or Class II is based on the degree of connection exhibited by their organic and inorganic components [11].

Physical interactions, including van der Waals forces, hydrogen bonding, and electrostatic forces, determine Class I, while covalent connections between inorganic and organic components determine Class II [11,88]. The sol-gel method is used to mix two distinct inorganic and organic components to produce Class I hybrid aerogels. For Class II, two hybridization approaches are generally used: first, as a precursor, organoalkoxysilanes are used, and the second method is the development of composites with structural supports or polymers. The co-precursor approach, which leverages the Si-C bond to insert organic moieties through an inorganic framework, may be used to enhance the strength of silica aerogels [11].

In the recent literature, several silica-based composite materials have been developed for various applications. The properties of aerogels have been enhanced by associating silica with different organic and inorganic components, rendering the resulting composite materials suitable for biomedical, environmental, and construction applications [87,89,90,91,92,93,94].

For instance, Maleki et al. [95] have obtained a scaffold, a silica-silk fibroin aerogel, with application in bone tissue and demonstrated its improvement properties (i.e., high specific surface area, high porosity with an impressive anisotropic micromorphology, and advanced mechanical behavior). They also proved the biocompatibility, attachment, growth, and proliferation of osteoblasts, specific bone tissue cells, and protein adsorption, such as hydroxyapatite, bone-type minerals due to the open porous microenvironment of the scaffold.

Another interesting example of a silica-based composite is offered by Jabbari-Gargari et al. [96], who reported the outstanding incorporation of aspirin, a model drug, on Trimethylchlorosilane silylated silica aerogel (TS-SA), a hydrophobic nanostructure. The researchers demonstrated the uniform adsorption of aspirin on the structure and surface of aerogel without drug degradation, and the tests showed a lower release rate of silica-based aerogel in comparison with pure drugs.

Differently, Cheng et al. [94] developed a composite made of silica aerogel and resin matrix that can be applied in dental restoration as a filler material. They investigated and demonstrated that the silica aerogel is uniformly mixed with the resin matrix, how the interaction between them is strengthened by hydrogen bonding, and how the interfacial bonding state is enhanced by interpenetration and mechanical interlocking. Also, they showed that in comparison with pure resin, the mechanical and antibacterial activity has been improved by adding a single filler, and the potential of silica-based material can be used as a new restorative material in the dental field.

Interesting emerging silica aerogel-based composites have also been designed in combination with diverse other materials, including but not limited to glass fibers [97,98], silica nanowires [99], titanium dioxide [100,101,102], ferric oxide [103], diatomite [104], reduced graphene oxide [105], sintered fly ash [106,107,108], biochar [109], melamine foam [110], paraffin [111,112,113], cellulose [105], polystyrene [114], polyurethane [115], polyimide [116,117], phenyl [118], C8/threonine [119], piperazine [120], amidoxime [121], and cinnamaldehyde [109].

An at-glance summary of various recently developed silica-based composites is offered in Table 3, highlighting the relevant properties of these materials and their potential applications.

Regardless, future interdisciplinary studies have the potential to generate optimized silica-based aerogel composites for use in numerous fields. By combining the advances in interconnected domains, such as chemical engineering, material sciences, nanotechnology, biomedicine, and environmental engineering, innovative solutions can be found for developing highly valuable silica-based aerogels with enhanced properties and improved functionality.

## 5. Applications of Silica-Based Aerogels

Due to their notable properties, such as uniformity and homogeneity, recognized at the beginning of 1980, aerogels were used for their unique primary application, like counters and particle detection, fuel capsules for inertial confinement fusion tests using direct drive or have been used as vacuum tubes for radiation detection and to sustain the high voltage wire with the cathode covering [128]. With the very quick development of new compositions, process techniques, and reinforcement strategies for structure and drying methods, aerogels, since the early 2000s, have become an impending material class with a very large potential in advanced technological applications [76].

**Figure 5:**
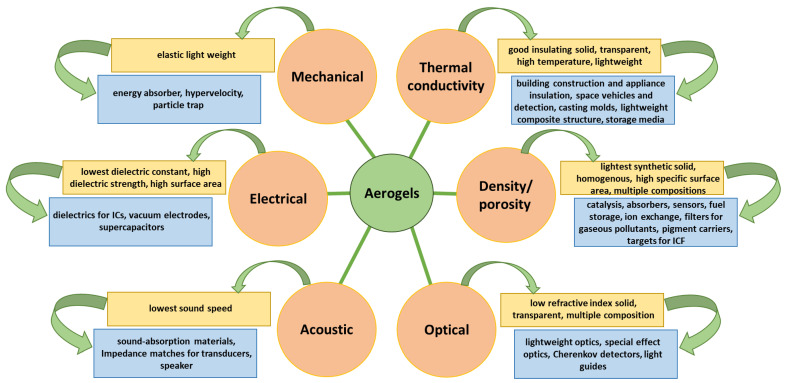
Correlation between properties, features, and applications of aerogel. Created based on information from [21,40,59,76].

Some particular properties of aerogels allow them to be used in several applications [40,59], as depicted in Figure 5 and detailed in the following subsections.

### 5.1. Building Applications

With a vast area of application in buildings, due to their excellent chemical and physical characteristics, such as low thermal conductivity and translucent appearance, aerogels are used in solar collector covers, roofs, windowpanes, materials for insulating in the construction of industrial and residential building, skyscrapers, ultralight structures, to give only a few examples. Even though the price is still high compared with conventional materials, silica aerogels are one of the greatest insulation materials. Moreover, aerogel made of inorganic silica is nonflammable and heat-resistant up to 1200 °C. As a result, it can be utilized as a flame-retardant material inside buildings. Furthermore, they can be used to insulate facades because of their dimensional stability and high-performance thermal insulation properties [5,76]. Silica aerogels have the benefit of enabling the simple integration of different compounds in their structure, such as particles, polymers, and fibers, to improve mechanical or thermal characteristics. For example, by reinforcing with polymers and modifying the silica backbone structure, the silica-based aerogels show enhanced parameters, like thermal insulation and density [55,129]. Another possibility is to create composites between silica aerogels and carbon foam, leading to materials with enhanced thermal insulation performance, especially when subjected to high temperatures from 100 to 600 °C [130]. Nano silica-based aerogels can also be effectively employed to fill the pores of other building materials. For instance, by uniformly assembling within the pores of lightweight carbon-bonded carbon fiber composites, the silica-based aerogel significantly improved its thermal insulating properties by diminishing the original material’s radiation and gas thermal conductivity [131].

In addition to being good heat insulators, aerogels are excellent sound insulators due to their acoustic qualities [5,76]. Perforated materials have very specific applications, such as ceiling tiles in theatres, concert halls, or auditoria, while foamed and fibrous materials are used in standard utilizations, for example, public buildings, commercial, residential, automotive, and other transportation, due to their easy installation and low cost. Mineral wool is a cost-effective material; however, it is unsuitable for use as a design or final cover because of concerns regarding health and environmental impact. For acoustical and architectural purposes, alternative materials such as perforated systems and foams are more suitable for internal and external designs, respectively. Aerogels can be used as a final coating when mixed with different materials because of their minimized dust-releasing performance [21].

Many strategies have been approached to produce novel materials based on aerogel, such as Bosagel, a BASF silica-based aerogel that has been advanced almost to market address beyond the past few years. The manufacturing of Basogel is a process of two steps, starting with sodium silicate, an inexpensive material, and sulfuric acid. Due to its low thermal conductivity, this material can help improve thermal insulation below atmospheric pressure, and it is used in regions equipped with heating and cooling equipment [5].

Besides Bosagel, several state-of-the-art materials may soon enter the market, as numerous recent research studies focused on manufacturing silica aerogels for building applications. Such novel materials include hollow glass fiber–silica aerogel composites with improved thermal insulation performance at high temperatures [97], silica aerogel foam concrete for use in construction [132], miscanthus fiber modified with hydrophobic silica aerogel for performant bio-lightweight concrete [133], and CaCl_2_·6H_2_O-silica aerogel composite phase change material for building energy conservation [134].

### 5.2. Aerospace Applications

Due to the low density and thermal conductivity values, silica aerogels are an attractive material necessary for insulation in large aerospace applications [135]. Long ago, NASA used thermal insulation monolithic silica aerogels [136]. For example, as part of the Pathfinder mission, silica aerogels were used for the first time as insulators on board the Mars Rover in 1997 to protect the Alpha Particle X-ray Spectrometer’s primary battery pack from the effects of low temperatures. Silica aerogels are also used to coat space suits in order to offer thermal insulation [42].

Even if they are used for thermal conductivity, silica aerogels have poor load-bearing capability and can be easily damaged. This fragile nature became helpful in various space missions, for example, obtaining cosmic dust particles at high speeds from a comet’s tail. The aerogel structure was employed to progressively slow them down to capture particles moving at high speed and preserve them for further study once they arrived on Earth [135].

This field benefits from the active scientific interest of researchers worldwide, with several studies being reported in recent years. Lately, new silica aerogel-based materials have been developed for aerospace applications, including fire-resistant polyimide-silica aerogel composites [116], TiO_2_-silica composite aerogel resistant to continuous-wave laser irradiation [101], and silica fiber-reinforced ceramic silica matrix composites filled with silica aerogel [137].

### 5.3. Agricultural Applications

A few divided particles are used in insecticide formulations as carriers that have insecticide effectiveness because they have abrasive and water-sorptive properties. The carriers consisted of silica aerogel and silica gel that were finely split. Silica aerogels are used as a powder form of insecticide. Silica aerogels are coated with 35–70% glycol (lower alkylene glycols) and ammonium fluosilicate 5% to reduce dustiness without modifying the characterizations of silica aerogels [138].

As a result of a large surface area and small particle size, aerogel powders can imbibe the protecting lipid layer of bugs, initiating the loss of body fluid of organisms and resulting in their death. Fumed silicon oxide, manufactured by silicon chloride combustion, has the same chemical composition as silica aerogels [80,138]. There are many insecticides based on silica aerogels for controlling the ants, mosquitoes, fleas, crickets, bedbugs, spiders, wasps, house flies, and other insects stored in homes, hotels, stores, schools, hospitals, theaters, or places where human and food are present. Silica aerogels are not toxic to mammals, are stable at high or low temperatures, and are effective even for species resistant to pesticides [138].

### 5.4. Environmental Applications

During synthesis, by controlling the hydrophobicity, a superhydrophobic behavior of silica aerogels can be obtained, making them a good material used efficiently in many environmental applications [139,140]. It was discovered that the superhydrophobic silica aerogels are superior absorbers of oils and diverse organic liquids. The organic liquids could be recovered again, and the aerogels may be reused as absorbents because of their exceptional absorption capacity [42,141].

Silica aerogel-based materials have been investigated as adsorbents of harmful heavy metals (e.g., Pb(II), Cu(II), and Pd(II)) [121,142,143], organic dyes (e.g., crystal violet, methylene blue, azophloxine, and acid green 25) [119,144,145,146], antibiotics (e.g., terramycin, cefixime, and ciprofloxacin) [147,148,149], and nanoplastics [150] from contaminated aqueous samples.

Moreover, they can also be involved in atmospheric CO_2_ capture, recovery, and transformation into more valuable products as an innovative method to reduce greenhouse gas emissions [151,152,153,154]. In this respect, they can be combined with different components, for example, wollastonite [155], bis(trimethylsilyl)hexane (BTMSH)/tetraethyl orthosilicate [156], and K_2_CO_3_ sorbents [157]. Such silica aerogel-based composites provide durable materials with high specific areas that show promise for CO_2_ sequestration and support fast carbonation reactions, demonstrating great potential for large-scale post-combustion processing in power plants [155,156].

### 5.5. Biomedical Applications

Biomedical applications of silica aerogels include carrier materials in medicine due to their high porosity, excellent stability, porous structure, easy functionalization of surface, adsorption properties, and substantial specific area. The loaded substance in aerogel can be delivered in a delayed or accelerated form by choosing an appropriate hydrophobic or hydrophilic aerogel. These characteristics make them suitable for high-performance drug carriers since they are biocompatible, biodegradable, and innocuous to humans [57,80,158,159].

Aerogel can work in synergy with other substances to produce composites with superior mechanical properties, a large porosity, and the lowest thermal shrinkage. Silica aerogel, due to its large specific surface area, has a high potential for use in dental areas. Resin composite and silica aerogel were mixed to obtain a filler material in medicinal dentistry with a matrix made of resin with antibacterial capabilities [94,160].

### 5.6. Other Applications

Due to their versatility and unique physicochemical features, silica aerogels have attracted increasing attention for a wide range of utilizations. Besides all the applications mentioned above, they can have other applications such as gas filters, encapsulation media, absorbing media, hydrogen fuel storage, interlayer dielectric, catalyst, catalyst carrier, sensor, templates for solar cells, Cerenkov radiation detectors, clothing, blankets, and more [59,80]. Due to high transparency and thermal insulation, silica aerogel has been utilized in solar-related fields, namely energy-saving buildings, solar absorbers, solar desalination, or, as a film, it was used as a dye for sensitized solar cells [40,77,161]. Another interesting application consists of aerogel “blankets”, for which commercial production started in the 2000s. Aerogel blankets are composed of a resilient and flexible material, a composite of silica aerogel and reinforcing fibers [4,42].

## 6. Challenges and Future Perspectives

Alongside all the medical, building, and aerospace applications, aerogels represent a novel material with a microstructure that can adsorb various contaminant types from water; silica-based aerogel, the lightest material, shows a benefit in water decontamination [44,162]. Compared with the mono-component advantageous properties, the functionalized aerogel composites show more advantages and new ways to be utilized [90,163]. However, some concerns about the large-scale utilization of silica-based aerogels have been observed. First of all, it is necessary to perform water decontamination studies on a real-life scale since most of them have been performed on a lab scale. Also, the environmental hazard is dependent on additional compounds introduced in silica aerogels [164,165]. Additional essential limitations that need to be examined are the possibility of secondary pollution by using the regeneration of developed adsorbents. Therefore, post-treatment studies are necessary to acquire an effective, ecological, and sustainable way to eliminate possible waste materials [165,166].

Other limitations of silica-based aerogels, such as their complex manufacturing process, mechanical fragility, and high processing costs, require particular attention. Given the scarcity of literature on financial estimation, a cost analysis must be thoroughly performed to help reduce future costs [165].

Despite the existence of numerous silica-based aerogels for biomedical applications, most of them are under development, necessitating further optimization of properties and synthesis methods. Moreover, to enter the market, silica-based aerogel formulations require in-depth testing and translation from in vitro/in vivo studies to clinical trials [57,167].

In terms of thermal insulation applications, producing silica-based aerogels at large with low-cost production is necessary for advancing the current technological maturity level. Also, the fragility and sensitivity at low pressure of silica-based aerogels impose the production of composites with improved mechanical strength, especially polymer-, carbon-, and organoalkoxysilane-reinforced [40].

Silica-based aerogels have only been considered for specific applications, especially due to their high cost and limited laboratory-scale production. However, these versatile materials might also gain track for use in mature industries, including automotive, oil, and gas, after their industrial production is more cost-effective [40].

## 7. Conclusions

The unique characteristics and diverse chemical compositions make aerogel a recognized state of matter. The categories, applications, and preparation methods of aerogel are diverse after being developed for ~80 years. In this paper, an updated review of silica aerogels, their preparation modes, and potential applications has been made to highlight their tunable nature and valuable potential. Reviewing the literature has confirmed that silica aerogel is one of the most popular and promising thermal insulation materials in recent decades.

However, aerogel currently has two more challenging points. The first is the cost, which is still considerably higher than that of conventional insulation materials, and the second is dust, which occurs during manufacture and is very difficult to remove. Nonetheless, future forecasts indicate that the unit price of aerogel will fall as a result of possible developments in material science and technology and that manufacturers will succeed in making panels and encapsulations more rigid to minimize dust, resulting in aerogel becoming more prevalent on the market and thus becoming a decent alternative to current traditional building insulation materials. The high potential for aerogels can be found mainly in translucency and possible transparency, as aerogels can offer significant energy savings in future windows, skylights, and other applications.

To conclude, silica-based aerogels can work in synergy with various materials, creating performant composites suitable for modern constructions, advanced aerospace applications, environmental solutions, and agricultural uses and opening the door to novel technological developments.

## Figures and Tables

**Table 1:** Advantages and disadvantages of different synthesis techniques for aerogel manufacture.

Synthetic Method	Advantages	Disadvantages	Aerogels Synthesized	References
Sol-gel	Easy control of nano architecture, cost-effective, and simple method	Aerogels are amorphous and time-consuming because of the aging step and the reaction to reach finalization; hydrolysis and condensation reactions are fast, having a meaningful impact on the structure	Hybrid aerogel, inorganic aerogel	[37,38,39,40,41,42]
Self-assembly	Crystalline aerogels; large and complex structures can be obtained	Uncontrolled self-assembly attends to aerogels with a complex structure	Graphene oxide aerogels, graphene-based aerogel, metallic aerogels	[37,43,44,45,46]
Emulsion	By predicting and optimizing, one may effectively regulate the ultimate particle size, spherical micro-aerogel	Removing the emulsifier is difficult, and aerogels are often impure	Polymer aerogel, inorganic oxide aerogels	[37,44,47]
Template	Complex structures with attractive resolutions; highly crystalline aerogels	Need to remove the template without damaging the structure	Functional aerogels	[37,44,48]
Epoxy	Mixed metal materials can be formed in nanoscale; controllable composition; control of aerogel properties	Aerogels are amorphous; time-consuming method	Oxide aerogels	[37,44,49,50]
3D printing	Accessible patterns; many complex structures can be created; fabrication requires only one step full device	Specific printing technologies, high cost, maintaining the solution viscosity; required post-processing (chemical and/or thermal process) to provide structural integrity and mechanical strength	Polymeric, hybrid aerogels	[37,48,51]

**Table 2:** Advantages and disadvantages of different drying techniques.

Drying Technique	Description	Advantages	Disadvantages	References
Low-temperature supercritical drying	This technique involves the replacement of synthetic solvent with supercritical carbon dioxide at a temperature of 31 °C	Lower temperatures; safe process; utilization of nonflammable solvents	Time consumption, organic solvents need to be soluble in supercritical CO_2_	[42,60,62]
High-temperature supercritical drying	This method uses a supercritical fluid, which is an organic solvent at a temperature nearly to 260 °C	High reaction rates of gelation and aging; relatively rapid discharge of the supercritical fluid	Flammability of the solvents, the structure may collapse because of the capillary pressure, which overcomes the strength	[42,53,60]
Ambient pressure drying	This process may start with room temperature evaporation and move on with increasing the temperature, which is below 200 °C	Safe, efficient elimination of pore fluid; simple, low-cost, and time-saving method	Serious shrinkage and cracking	[7,42,63,64,65]
Freeze-drying	The freeze-drying method involves low temperature, between −50 and −83 °C, and a vacuum pressure, 5–30 Pa	Defeats the negative impact of capillary pressure; environmentally friendly; less expensive than supercritical drying; prevents shrinkage	It can create cracks; it requires an aging step for full polymerization and condensation, solvent exchanging with a low coefficient of thermal expansion	[40,66,67]
Microwave-drying	This method involves microwave irradiation without implying high temperatures or high pressures	Low cost, the drying process can be controlled, and no costly equipment is required	Duration of a process is limited by the progress at which heat enters the body, increasing energy consumption, not used industrially, large pores formation	[61,68,69,70]
Vacuum drying	This technique assumes the application of vacuum at ambient temperatures	Energy and time-saving	Not used industrially; large pores formation decreases the amount of surface area on the solid material	[61,71]

**Table 3:** Examples of different silica-based materials, their properties, and applications.

Composition	Properties					Applications	Refs.
	Porosity (%)	Surface Area (m^2^g^−1^)	Pore Volume (cm^3^ g^−1^)	Density (g/cm^3^)	Other Properties		
Monolithic alumina/Silica aerogels	-	120.6–575.5	0.7	0.243–0.249	Compressive stress 1.78 MPa; Elastic modulus 65.6 MPa	Thermal insulation, catalysis	[122]
Magnetic iron oxide/Silica aerogel	-	310.8–411.0	0.85–1.12	-	Adsorption capability 95.8%	Rhodamine B adsorption	[123]
Carbon nanostructures/Silica aerogel	93.9–95.2	293.7–465.4	12.5–12.8	73–76		Adsorption of organic pollutants	[124]
Chitosan/Silica aerogels	83–85	139.76	0.149–0.182		Water contact angle of 142°; Thermal conductivity 0.024–0.034 Km^−1^K^−1^ at room temperature	Energy conservation of building insulation, environmental protection	[116]
Silica aerogel/Chitosan/Gelatin loaded with quercetin	-	313	-	0.82 m^3^	Average pore diameter 9.36 nm	Drug carrier	[125]
Silica/Chitosan composite aerogels	-	800–1100	2.5–4.0	0.144–0.199	Young’s modulus 0.66–22.61 MPa	Bone tissue engineering	[126]
Silica-silk fibroin bioaerogel	91–94	433–531		0.077–0.115	Young’s modulus 4.03–7.3 MPa	Bone regeneration	[95]
Trimethylchlorosilane silylated/Silica aerogel/Aspirin	-	495–580	2.6–3.1	-	Contact angle od TS-SA 152°; Loaded drug dissolve 35% ± 4 and 80% ± 4 in 0.1 N HCl	Drug delivery	[96]
Silica nanowires/Silica aerogels	93.6–93.8	236–286	0.19–0.21	0.118–0.121	Compressive strength 1.379 MPa; Contact angles 140°	Thermal insulation	[99]
TiO_2_/Silica aerogel	87.71	491.0613	-	0.3968	Remain intact at laser irradiation with power of 7 kWcm^−2^	Photocatalysis and optical limiting	[101]
SiO_2_/Fe_2_O_3_ composite aerogels	-	565–705	-	0.04–0.18	Thermal conductivity 0.026 W/(m K); Compressive strength 0.40 MPa	Thermal insulation enhancement	[103]
Graphene oxide/Silica aerogel	95–96	863.4–913.1	3.54–4.17	~0.10	Thermal conductivity ∼ 0.023 W/m/K	Thermal insulation applications	[127]
Silica aerogel/Melamine foam	-	62.8–601.2	-	0.014–0.091	Thermal conductivity 0.027–0.072; Oil adsorption capacity 36 g·g^−1^	Oil/water separation and self-cleaning	[110]
Polystyrene/Silica aerogels	80.45–83.13	509–999	-	0.218–0.251	Elastic modulus 5.17–29.35 MPa; Thermal conductivity 19.4 mW m^−1^K^−1^	Construction materials with high insulation properties	[114]
Polyimide/Silica aerogel	-	748.8–800.6	2.993–3.053	0.061	Thermal conductivity 0.0216 Wm^−1^K^−1^)	Aerospace applications	[116]
Polyimide/silica aerogels	-	356	3.27	0.196	Specific modulus, 54.45 kN·m·kg^−1^; Thermal conductivity 0.02080 Wm^−1^K^−1^	Thermal insulation	[117]
Phenyl/Silica aerogels	-	-	1.14–2.86	0.19–0.23	Thermal conductivity 0.0241–0.0283 Wm^−1^K^−1^	Thermal insulation and self-cleaning	[118]
C8/Threonine/Silica aerogels	-	596.20	0.511	-	Adsorption capacity for azophloxine 152.43 mg/g and for methylene blue 274.30 mg/g	Adsorption of cationic and anionic dyes	[119]
Piperazine/Silica aerogel	-	1124	2.701	-	Remain intact at laser irradiation with power of 7 kWcm^−2^	Direct air capture of CO_2_	[120]
Amidoxime/Silica aerogels	-	286.45–401.43	3.7677–7.5250	-	Adsorption capacity for Pb(II) 598.05 mg/g and for Cu(II) 534.10 mg/g	Adsorbent for heavy metal ions	[121]
